# Physical and thermomechanical characterization of unidirectional *Helicteres isora* fiber-reinforced polylactic acid bio-composites

**DOI:** 10.1038/s41598-024-65591-3

**Published:** 2024-06-26

**Authors:** Prashantha Acharya, Dayananda Pai, N. H. Padmaraj, G. T. Mahesha

**Affiliations:** Department of Aeronautical and Automobile Engineering, Manipal Institute of Technology, Manipal Academy of Higher Education, Manipal, Karnataka 576104 India

**Keywords:** Helicteres isora, Polylactic acid, Polymer matrix composites, Tensile properties, Flexural properties, Thermogravimetry, Mechanical engineering, Composites

## Abstract

Identifying novel cellulose fiber bio-composites has become a vital initiative in the exploration of sustainable materials due to increased global concern for the environment. This growing focus on eco-friendly materials has gathered significant attention in recent years. The current investigation deals with one such material, *Helicteres isora* reinforced Polylactic acid composites. Surface chemical treatment of fiber is one of the most effective methods to modify the hydrophilic fiber to increase its compatibility with the polymer matrix. Sodium hydroxide was used as a pre-treatment chemical to remove any impurities from the fiber surface. Pre-treated fibers were treated with Methacryl silane and Potassium permanganate solution to chemically modify the fiber surface. Density, void content and water absorption behavior of the composites were analyzed as per the standard procedure. Tensile and flexural tests were conducted to evaluate the mechanical strength, modulus, and flexibility of the unidirectional composites. Thermogravimetric and differential thermal analyses were performed to investigate the thermal stability, melting behavior and degradation profiles of prepared composites. A study of failure mechanisms and morphology of the fractured surface through photographs and SEM images revealed fiber splitting and delamination as the dominant reasons behind the failure of composites under tensile loading. Silane-treated *Helicteres isora* fiber-reinforced Polylactic acid composite exhibited lower water absorption and higher tensile strength than its counterparts. Untreated fiber composite showed maximum flexural strength among the tested composites. By collectively evaluating the results of the tests and properties of the composites, silane-treated fiber-reinforced Polylactic acid composites stands out as the most favorable choice.

## Introduction

Composite materials, characterized by their exceptional properties and diverse applications, consist of two or more chemically distinct components: the matrix and the reinforcement. Typically, the reinforcement, known for its superior rigidity, complements the matrix, providing a homogeneous structure^1,2^. Natural fibers, recognized for their technological and ecological advantages, are emerging as a prime substitute for synthetic fibers in the composite industry^3^. Research and development efforts have revealed the advantages of natural fibers, particularly in industries such as automobiles, construction, and aerospace. Natural fiber-reinforced polymer composites prove advantageous due to their cost-effectiveness, low density, minimal Carbon dioxide (CO_2_) emissions, nonabrasive nature, low energy consumption during production, and reduced health risks^4,5^. In a comparative analysis, it was documented that substituting 30% of glass fibers with 65% hemp fibers resulted in an energy savings of 50,000 MJ^6,7^. While natural fibers offer various advantages, they present challenges such as compatibility issues with the matrix, limited resistance to moisture, dimensional instability, and the possibility of aggregate formation in the fabrication process^8–10^. Plant fibers possess effective thermal and acoustic insulation characteristics owing to their inherent hollow and lignocellulosic structure. Although the mechanical properties of plant fibers may be lower when compared to synthetic fibers, these can be improved through appropriate surface modifications. Surface modification of plant fibers is comparatively easier when compared to inert synthetic fibers such as glass, aramid, and carbon fibers^11,12^. Various high-cellulose natural fibers found in rural areas are commonly used by rural communities to produce different types of ropes and household items. These fibers have the potential to be further developed for numerous engineering and other applications. One such plant, *Helicteres isora*, yields a significant amount of bast fibers with a cellulose content up to 75%. These fibers can serve as an effective reinforcement in polymer composites. Despite its potential, this fiber is underutilized as an engineering material, with limited applications^13,14^. It is proposed that value addition to locally abundantly available such natural materials is possible by processing them into alternative materials for suitable applications.

Researchers have explored *Helicteres isora* fibers as reinforcement with polymers such as epoxy and polyester and characterized them for mechanical, thermal and physical properties. While the use of *Helicteres isora* fibers in such synthetic matrix composites showed promising results for various engineering applications, there were some limitations concerning biodegradability and the dependence on synthetic-based matrices^14–16^. For applications that prioritize sustainability, the use of non-biodegradable and synthetic-based composites presents significant environmental concerns. Manufacturers are increasingly looking for materials that not only perform well but also have minimal environmental impact. Such a bio-based and bio-compostable polymer, Polylactic acid (PLA), one of the most prevalent bio-based polymers, is notable for its favorable mechanical properties, processability, biodegradability, and biocompatibility. As a result, it finds widespread applications in various manufacturing sectors, including but not limited to packaging materials, biomedical materials, and thin-film materials^17,18^. Over the past two decades, there has been a growing trend of substituting man-made fibers with natural fibers owing to their biodegradability and environmentally friendly characteristics^19^. High-stiffness biocomposites might be beneficial in lightweight structural components of an automobile. Research indicates that employing natural fiber composites can potentially result in a 20% reduction in costs and a 30% reduction in the weight of vehicle components^20^.

Tensile characterization is essential for evaluating the strength and elongation behavior of materials under applied forces. The ultimate tensile strength, modulus, and elongation at break were analyzed by subjecting the composites to tensile loading. Flexural characterization, on the other hand, explores the material's resistance to bending. This property is crucial in applications where the material experiences bending loads, such as in structural components or load-bearing elements. Thermal characterization plays a pivotal role in understanding how materials respond to temperature variations. Thermogravimetric Analysis (TGA) enables the assessment of thermal stability and degradation, while Differential Thermal Analysis (DTA) provides information about the material's heat flow and phase transitions. Understanding the thermal behavior helps in predicting the material's performance in various environmental conditions and contributes to the selection of such eco-friendly materials for real life applications^21^.

The present study involves the characterization of unidirectional *Helicteres isora* fiber-reinforced PLA composites. A series of surface chemical modifications were carried out on the untreated fiber to enhance the bond between the fiber and the matrix in composite materials. This process involved the application of three distinct solutions: Alkali, Silane, and Permanganate. Each solution served as a treatment to modify the surface characteristics of the raw fiber, aiming to optimize its interaction and compatibility with the matrix in the composite material. The purpose of these modifications is to improve the overall performance and properties of the composite by building a stronger and more effective bonding between the reinforcing fibers and the matrix^22–24^. The study introduces a novel unidirectional composite derived from PLA reinforced with less explored high cellulose, high-strength natural fiber extracted from *Helicteres isora*, abundantly available in western ghat forests in India. Fabrication of the developed composite was carried out using an innovative process that combines solution casting and compression molding with heating. The fabrication method involves preparing prepregs of fiber mats coated with PLA solution, which are then meticulously stacked and compressed under controlled pressure and temperature. This approach not only enhances the fiber-matrix adhesion but also optimizes the mechanical and thermal properties of the composite, making it a promising material, which is totally biodegradable for advanced engineering applications, particularly in automotive interiors and lightweight structural components. Density, void content, water absorption behavior, Tensile and flexural properties, along with failure surface morphology using Scanning Electron Microscopy (SEM) images, and thermal properties of the composites were studied. This study assessed the thermal behavior of *Helicteres isora* fiber-reinforced PLA composites through TGA and DTA techniques.

## Materials and methods

**Figure 1:**
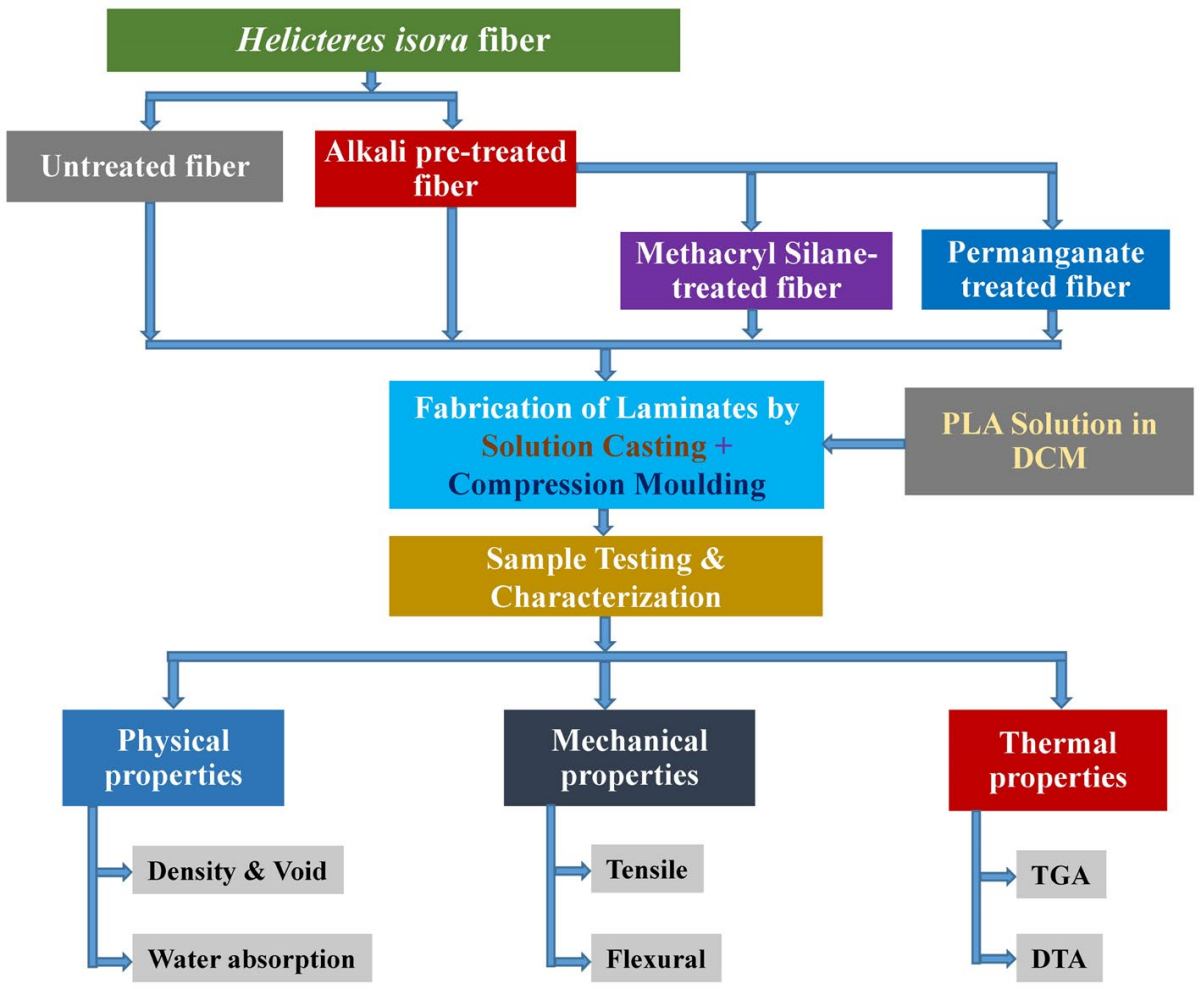
Flow diagram showing the methodology.

The fabrication of untreated and chemically treated *Helicteres isora* fiber-reinforced PLA composites was done by the combination of two processes. The solution casting technique was used to prepare prepregs of *Helicteres isora* fiber mats coated with PLA, followed by compression moulding with a heating facility. The detailed steps or stages of the methodology are presented visually in Fig. 1, and each step is discussed in detail and explained in subsequent sections.

### Materials

Raw bast fibers from the *Helicteres isora* plant were procured from the local forest in southwestern coastal India, and the clean fibers from well-grown branches were extracted by water retting for 15 days, which is also followed in the published work^13^. Retted *Helicteres isora* fibers were carefully separated and cleaned with running water until the visible impurities were removed. The diameter of *Helicteres isora* fiber was in the range of 90 to 120 microns as per the measurements done using the toolroom microsope (Mitutoyo TM-510). The microfibrillar ngle of the fiber is 20°–26°, and the length-to-diameter ratio of ultimate cells is 99^14^. With a fiber Further, the fibers were treated with chemicals for surface modifications. Alkali Sodium hydroxide (NaOH) is used as a preliminary treatment and further treated with Methacryl silane (Silane) and Potassium permanganate (PP) solutions.

In the current study, Ingeo biopolymer, 2003D grade PLA is used as matrix material, a plant-based bio-compostable thermoplastic supplied by Natur Tec India Pvt. Ltd, Chennai, India. PLA is the most widely used bio-polymer in composite applications because of its better physical and mechanical properties than other starch-based thermoplastics^25^. Detailed specification of PLA is given in Table 1 as provided by the supplier.

**Table 1:** Specifications of PLA 2003D supplied by Natur Tec India Pvt. Ltd.

Sl. No	Particulars	Value	Test standard followed
1	Specific gravity	1.24 g/cm^3^	ASTM D782
2	Tensile strength	60 MPa	ASTM D882
3	Elastic modulus	3.5 GPa	ASTM D882
4	% Elongation	6.0	ASTM D882
5	Heat distortion temperature	55 °C	ASTM E2092
6	Melting temperature	210 °C	–

### Chemical treatments

Raw *Helicteres isora* fibers were made to undergo a series of treatments to enhance their properties. Initially, they were immersed in a 5% w/v NaOH aqueous solution to eliminate impurities on the fiber surface, with a soaking duration of 60 min^13^. Following the NaOH treatment, two separate chemical treatments were performed using Silane and PP solutions. One batch of NaOH pre-treated *Helicteres isora* fibers was treated with a 2% v/v Methacryl silane solution in a mixture of ethanol and deionized water (ratio 60:40) for 60 min^26^. Another batch was treated with a 0.5% w/v aqueous solution of PP for 3 min^27^. After each chemical treatment, the fibers were rinsed thoroughly with distilled water until achieving a neutral pH of the rinsed water. These untreated, NaOH, Silane and PP solution-treated fibers were used to prepare individual composites with PLA as a matrix.

### Fabrication of *Helicteres isora* fiber-reinforced PLA laminated composites

The combined method of fabrication, which comprises solution casting to impregnate PLA into *Helicteres isora* fiber mat followed by compression moulding technique, is used for preparing composite specimens. Combining solution casting and compression molding techniques unveiled an adaptable and novel approach to fabricating *Helicteres isora* fiber-reinforced PLA composites. The stages of fabrication are explained in detail in the following sections.

#### Preparation of prepregs using solution casting technique

**Figure 2:**
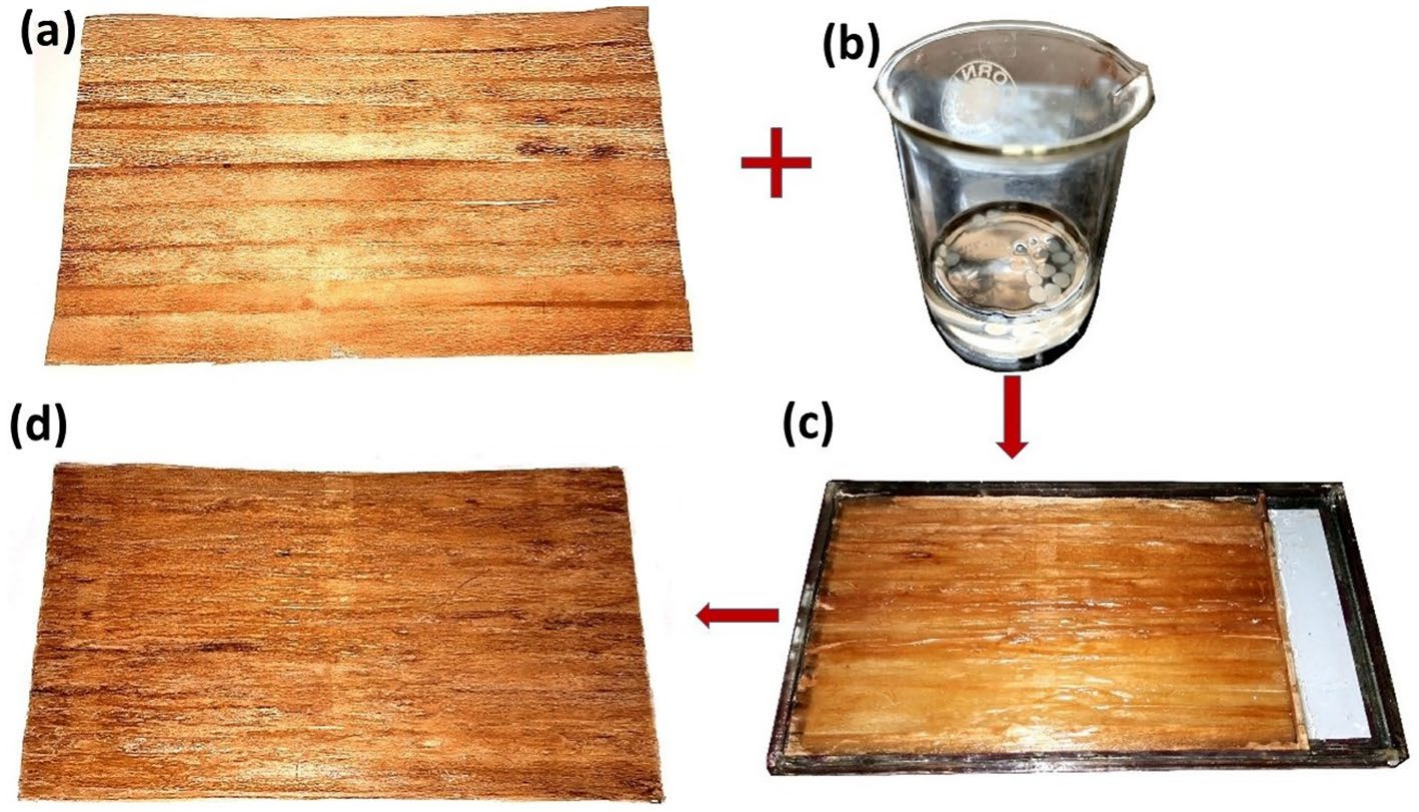
Solution casting process: (**a**) Fiber mat prepared, (**b**) PLA solution in MDC, (**c**) Solution casting in a glass tray and (**d**) Solution-casted PLA resin-coated *Helicteres isora* fiber mat.

The mat of *Helicteres isora* fiber was prepared by gluing the procured fiber layers side by side, as shown in Fig. 2a, with the dimension of 160 mm × 250 mm for processing with PLA solution. The average value of fabric density for the formed fiber mat was found to be 75–80 GSM. The PLA solution was prepared using dichloromethane (methylene-di-chloride or MDC) by blending a specific quantity of PLA granules into the MDC solvent as in Fig. 2b^28^. This resulting liquid PLA solution served as liquid resin for preparing matrix-coated *Helicteres isora* fiber mats with a 50% fiber volume fraction^16^. A glass tray was used for solution casting to impregnate or coat *Helicteres isora* fiber mats with PLA, as shown in Fig. 2c. The desired amount of PLA solution was poured and spread evenly in the glass tray and the fiber mat was placed on it. One more layer of PLA solution is spread evenly on the fiber using a soft brush, allowing it to cure. While curing, MDC evaporates completely and the PLA coats onto the fiber mat, which is shown in Fig. 2d. Similarly, multiple fiber mats were prepared to fabricate untreated and chemically treated *Helicteres isora* fiber and PLA laminated composites.

Along with the prepreg preparation, a clear PLA film of a thickness of 100 microns was prepared using the solution casting technique, and the same has been tested for Tensile and Thermal properties.

#### Preparation of laminates using compression moulding

**Figure 3:**
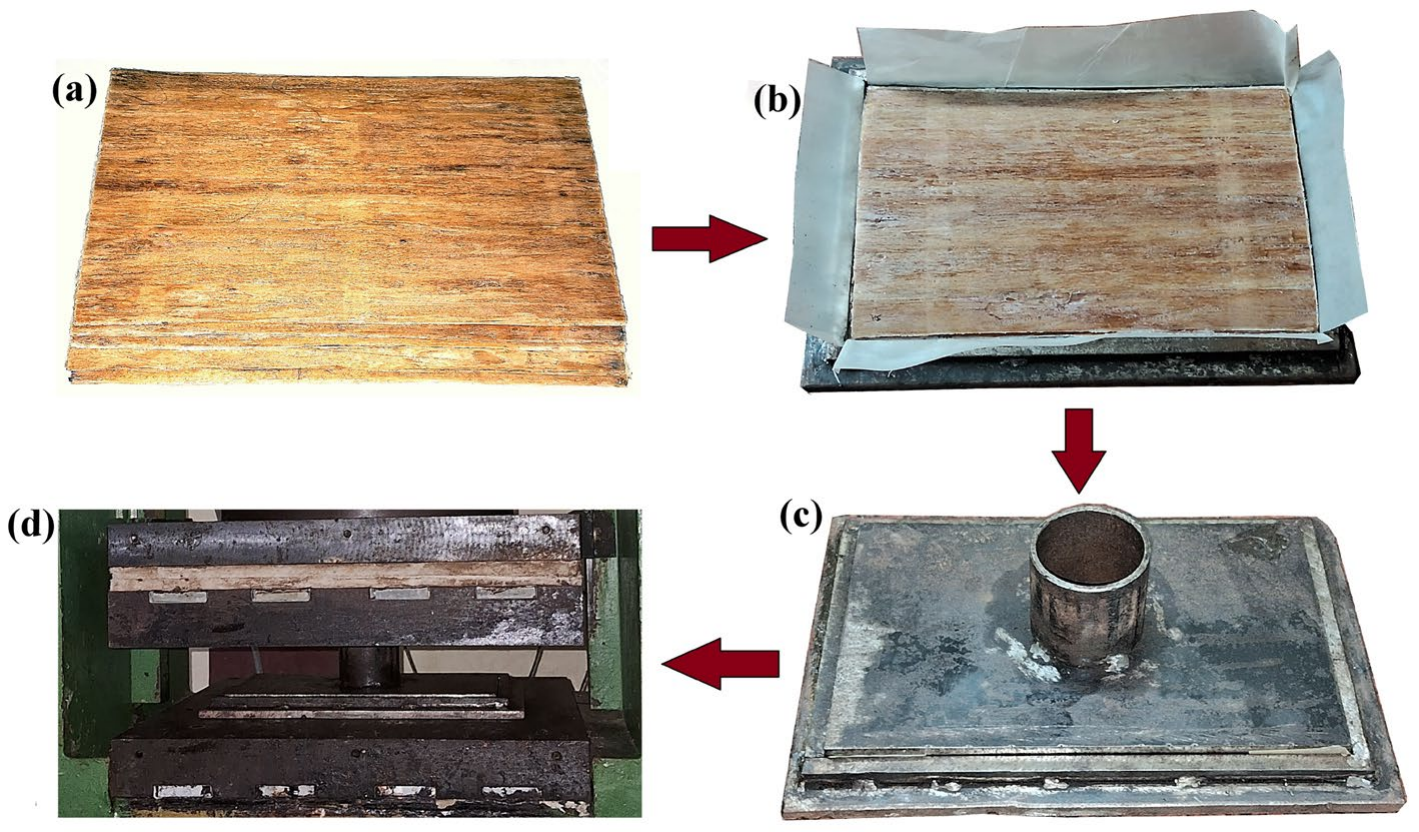
Process of preparing composites: (**a**) Prepregs of *Helicteres isora* fiber mats, (**b**) Prepregs in a greased steel mould, (**c**) Mould with top cover and (**d**) Mould placed in compression moulding machine under pressure.

**Figure 4:**
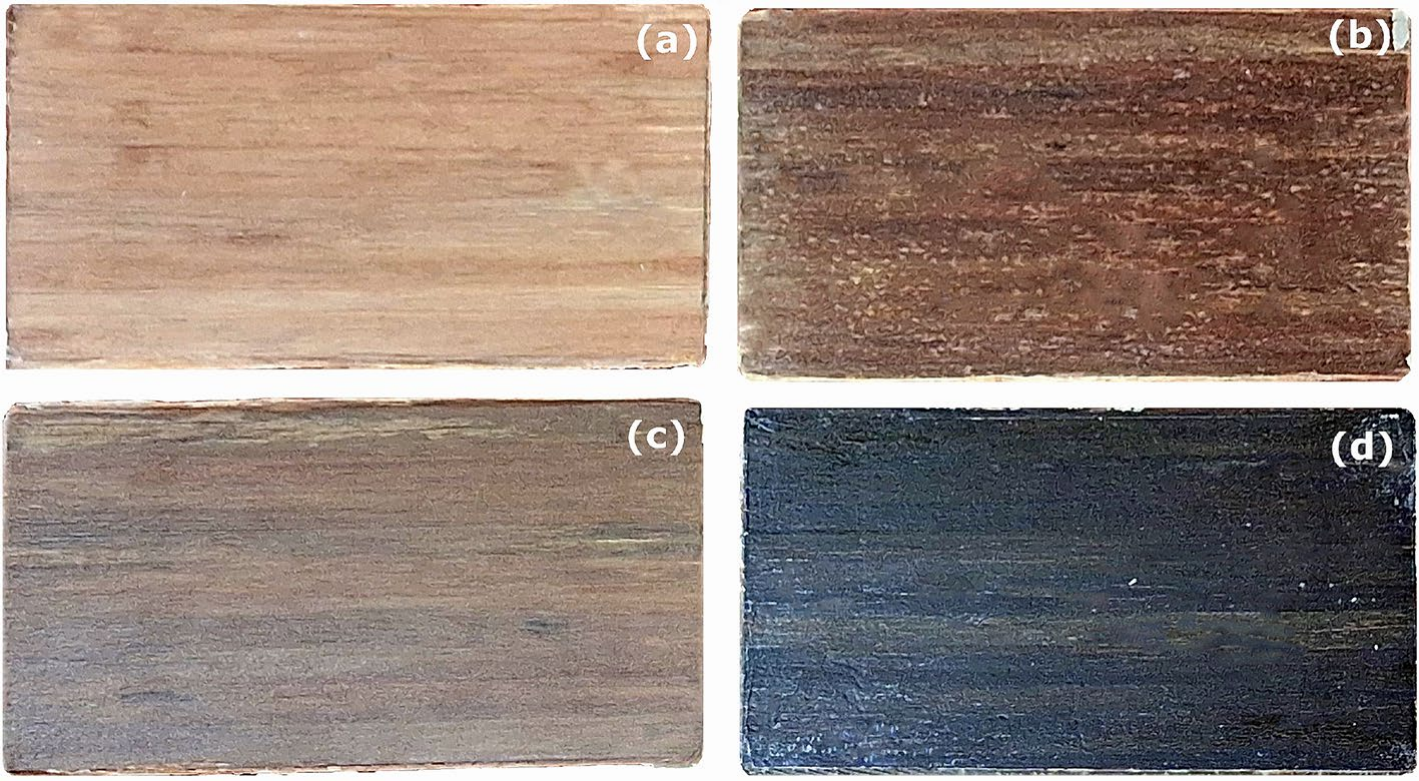
Composite laminates prepared: (**a**) UT, (**b**) AT, (**c**) ST and (**d**) PT composites.

Prepregs of *Helicteres isora* fiber mats prepared were stacked and placed in the steel mould, as shown in Fig. 3a,b. The steel mould with fiber mats as in Fig. 3c was kept under a pressure of 2 MPa in the compression moulding machine (Modern Hydraulics Compression Moulding machine), which is demonstrated in Fig. 3d, maintaining at a temperature of 150 °C for 15 min^29^. Subsequently, the laminate was left within the mold, still under pressure, to cool and cure for 24 h. Later, the laminate was removed from the mould and stored at room temperature with dead weight for another 24 h to cure completely. The fabricated specimens in this method are shown in Fig. 4. Untreated, NaOH, Silane and PP-treated fiber-reinforced PLA composites are abbreviated as UT, AT, ST and PT respectively, and the same notations are used in all upcoming sections.

#### Physical properties of the composites

The experimental density of the individual specimens was determined by measuring the weight and volume of the specimen. The laminates were cut into specimens of 20 mm × 20 mm size to conduct physical properties tests. The theoretical density, on the other hand, is computed using the rule of the mixture (ROM), as shown in Eq. (1), and the void fraction is calculated using the theoretical and actual densities of the composites. The void content of the composite prepared was calculated using Eqs. (2) and (3), as per ASTM-D2734.

1 $${\uprho }_{\text{c}}={\uprho }_{\text{f}} f+{\uprho }_{\text{m}}\left(1-f\right)$$

where: ρ_c_ is the density of composite, ρ_f_ is the fiber density, ρ_m_ is the matrix density, and *f* is the fiber volume fraction.

2 $$T = \frac{100}{\left(\frac{R}{D}+\frac{r}{d}\right)}$$

where: T is the theoretical density, R is the matrix weight %, D is the density of matrix material, r is the fiber weight %, and d is the density of fiber

3 $$\text{V }= \frac{100\left({\text{T}}_{d}-{\text{M}}_{d}\right)}{{\text{T}}_{d}}$$

where: V is the void content, volume %, T_d_ is the theoretical composite density and M_d_ is the experimental density.

The water absorption ability of the composite specimens was analyzed using the immersion method. The specimen was immersed in distilled water, and the weight gain was recorded for predefined intervals of time. Weight gain by the specimen is plotted against the square root of time in seconds. Equation (4) illustrates the relation between mass gain and diffusion coefficient calculated using Eq. (5)^30^.

4 $${M}_{t}=\frac{2{M}_{\infty }\sqrt{D}\sqrt{t}}{\sqrt{\pi } l}$$

5 $$D=\frac{\pi {l}^{2}{\theta }^{2}}{4 {{M}_{\infty }}^{2}}$$

where: M_t_ is the mass gain at time ‘t’ (g), M_∞_ is the mass gain at t = ∞ in (g), t is the time (s), l is the half thickness of the specimen (m), θ is the slope of M_t_ vs $\sqrt{t}$ from the graph.

#### Mechanical testing of specimens

**Figure 5:**
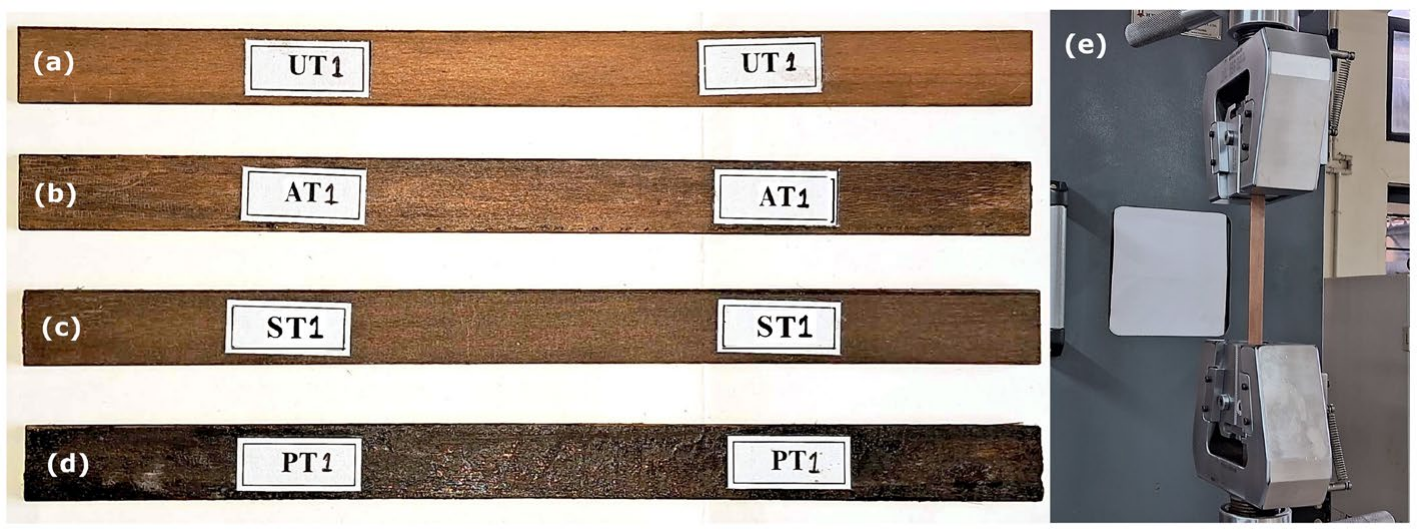
Tensile test specimens of (**a**) Untreated, (**b**) Alkali-treated, (**c**) Methacryl silane-treated and (**d**) Permanganate-treated *Helicteres isora* fiber-reinforced PLA composites, and (**e**) Tensile test setup.

Specimens for tensile, flexural and void fraction or density measurement were cut from the laminates as per the established testing standards. Tensile specimens (of dimension 200 mm × 15 mm × 2 mm) were cut as per ASTM D 3039 test method as in Fig. 5a–d. The tensile test was conducted using a BiSS universal testing machine with a load cell capacity of 50 kN, and a specimen-mounted setup is shown in Fig. 5e. The test was conducted at a speed of 2 kN per minute for each specimen with a data sampling rate of 30 per second.

**Figure 6:**
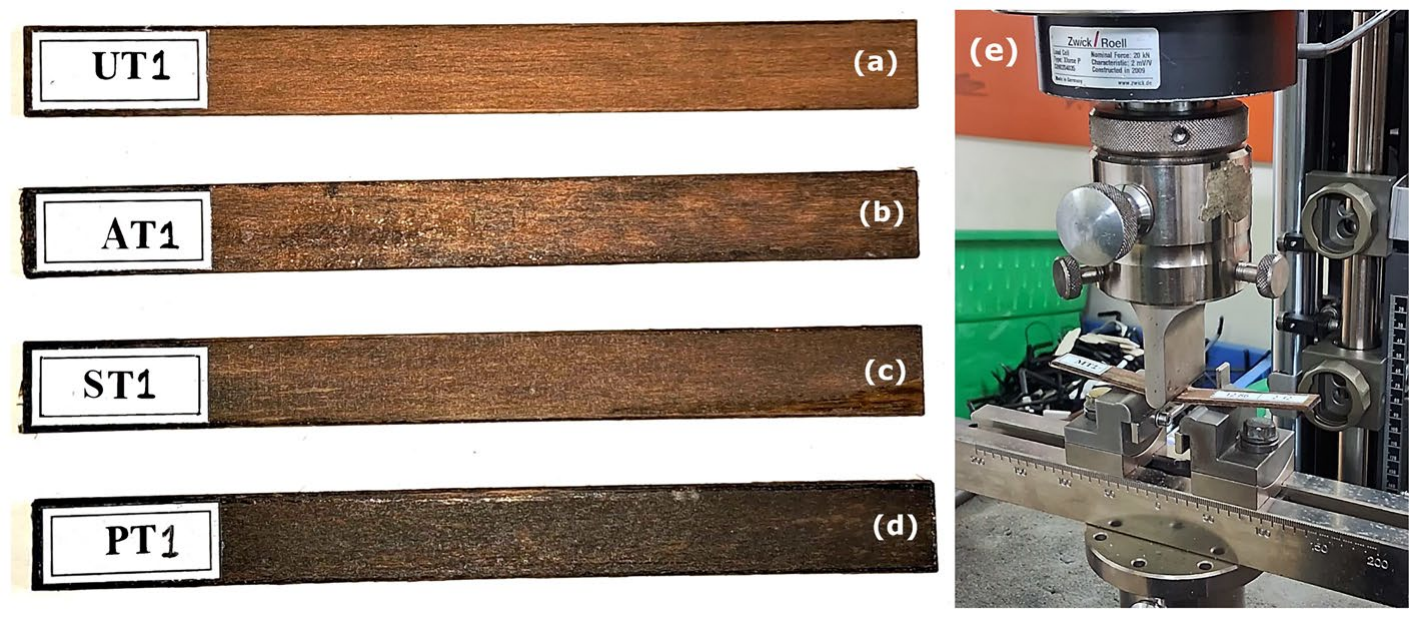
Flexural test specimens of (**a**) Untreated, (**b**) Alkali-treated, (**c**) Methacryl silane-treated and (**d**) Permanganate-treated *Helicteres isora* fiber-reinforced PLA composites, and (**e**) Flexural test setup.

Flexural specimens ( of dimension 127 mm × 12.7 mm × 2 mm) were cut as per ASTM D790 as shown in Fig. 6a–d. The flexural test was conducted using a Zwick Roell universal testing machine, a setup for polymer testing as shown in Fig. 6e, the test speed kept was 1 mm/min.

#### Thermal characteristics

Thermogravimetric analysis (TGA) and Differential thermal analysis (DTA) were performed on each composite using MAS-5800 instrument with a heating/cooling rate of 10 °C/min. TGA and DTA curves of raw *Helicteres isora* fiber, neat PLA specimen, UT, AT, ST and PT composite specimens are discussed in Sect. 3.3.

## Results and discussion

### Density, void content and water absorption behaviour of composites prepared

Theoretical densities of UT, AT, ST and PT composites based on ROM were calculated. The void fraction is calculated based on these values of theoretical densities of the composites. The densities and void fractions in percentages of the composites are shown in Table 2. The average void fraction of the tested specimen was less than 5% in all the cases. Least density was found in UT composite specimen (1.204 ± 0.012 g/cm^3^) among its counterparts, and PT specimen showed the least void content (3.45 ± 1.130%), followed by ST specimen (3.96 ± 0.840%). Due to the shrinkage of fiber upon alkali treatment, the density of the composite made from alkali-treated fiber increased. In permanganate treatment, the fiber density is reduced due to the rough surface formed, resulting in the reduced density of the composite made with permanganate-treated fiber^31^.

**Table 2:** Density and void content of specimens.

Composite	Theoretical density (g/cm^3^)	Density measured (g/cm^3^)	Void content %
UT	1.260	1.204 ± 0.012	4.41 ± 0.950
AT	1.278	1.219 ± 0.013	4.62 ± 1.000
ST	1.272	1.222 ± 0.011	3.96 ± 0.840
PT	1.259	1.216 ± 0.014	3.45 ± 1.130

**Figure 7:**
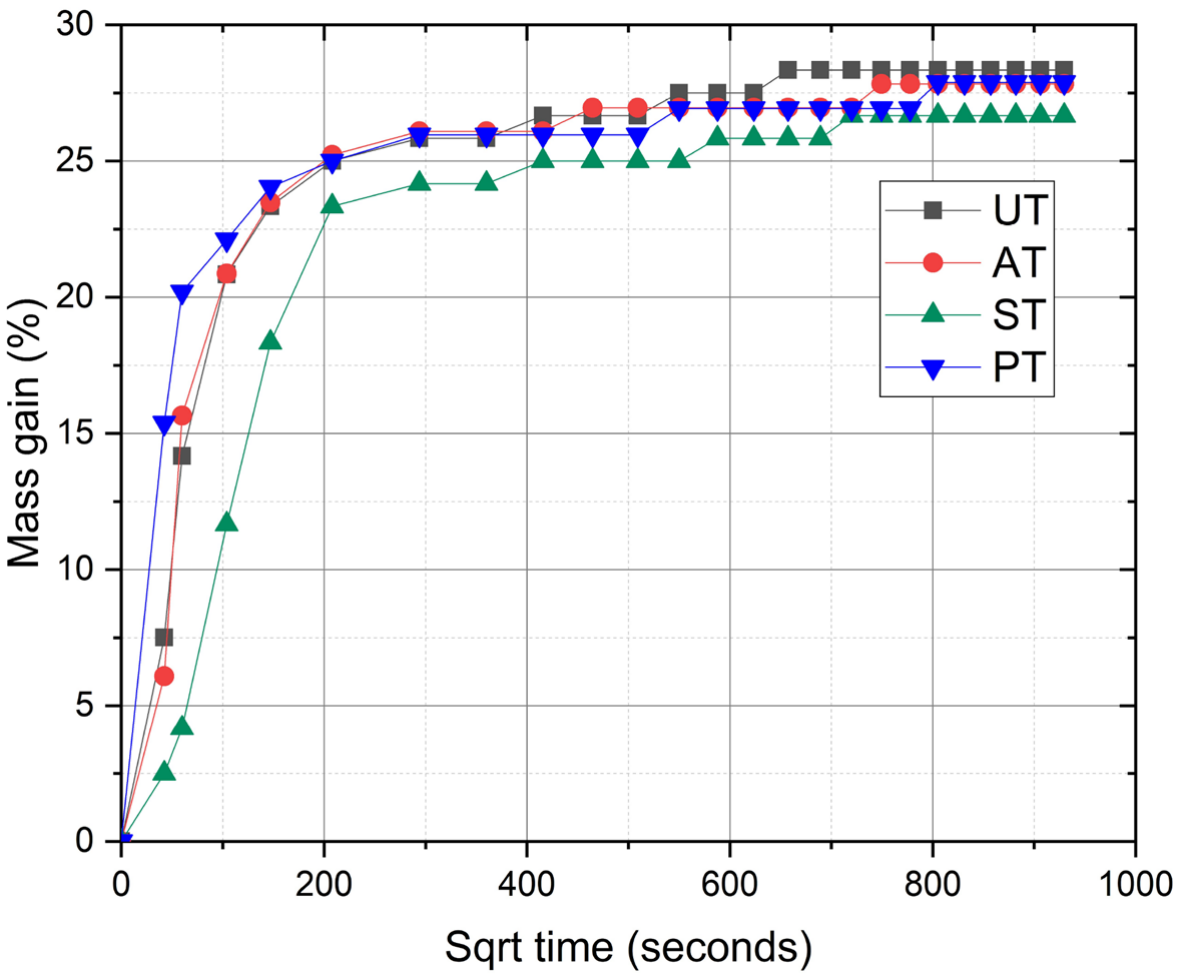
Water absorption tendency of UT, AT, ST and PT composite specimens.

The water absorption tendency of the specimens is plotted in the graph as mass gain % versus square root of time in seconds, as shown in Fig. 7. The diffusion coefficients of specimens are listed in Table 3. The diffusion coefficient plays a crucial role in understanding and predicting the water absorption behavior of polymer composites. It is a quantitative measure that reflects the rate at which water molecules move within the composite material. Understanding the diffusion coefficient allows for the prediction of water uptake over extended periods, which is essential for applications where long-term durability and dimensional stability are of paramount importance^32^. A high content of cellulose is responsible for fibers’ significant moisture absorption. Abundantly available polar hydroxyl groups within the cellulose crystalline region impart hydrophilicity to the natural fibers^33^. In the current work, the constituent analysis of raw *Helicteres isora* fiber using the gravimetry method revealed a remarkable cellulose content of up to 70% in the raw fiber, which is comparable to 74% cellulose content in raw *Helicteres isora* fiber as claimed in the published literature^14^. Silane treatment significantly reduces the fiber's susceptibility to water ingress by eliminating surface hydroxyl groups by chemically modifying the fiber surface, converting hydrophilic hydroxyl groups into water-resistant bonds^34^. The water absorption ability of the material also depends on the void content present in the material. Water absorption in the specimens UT and AT with higher void content was more compared to lower void content specimens ST and PT. As the void content decreases due to effective fiber treatment, the diffusion coefficient of the composite also tends to decrease. This is because the presence of voids facilitates water uptake and transport within the material. When void content is minimized, the water molecules encounter more resistance as they traverse the composite, leading to a lower diffusion rate. Consequently, the composite exhibits reduced water absorption, which is advantageous for maintaining its mechanical integrity and retarding degradation of the material over time. The present study on water absorption of the composites revealed that the silane-treated fiber-reinforced composite has shown reduced water absorption and a slower rate of water absorption than other composites.

**Table 3:** Diffusion coefficient of composites.

Composite	Mass gain at ‘t = ∞’ M_∞_ (g)	Half thickness of specimen *l* (mm)	Slope of M_t_ vs $\sqrt{t}$	Diffusion coefficient D (mm^2^/sec)
UT	28.33	1.11	9.867	3.260 × 10^−5^
AT	27.83	1.05	10.037	3.128 × 10^−5^
ST	26.67	1.18	7.817	2.609 × 10^−5^
PT	27.88	1.10	8.977	2.737 × 10^−5^

### Mechanical characterization

#### Tensile test

**Figure 8:**
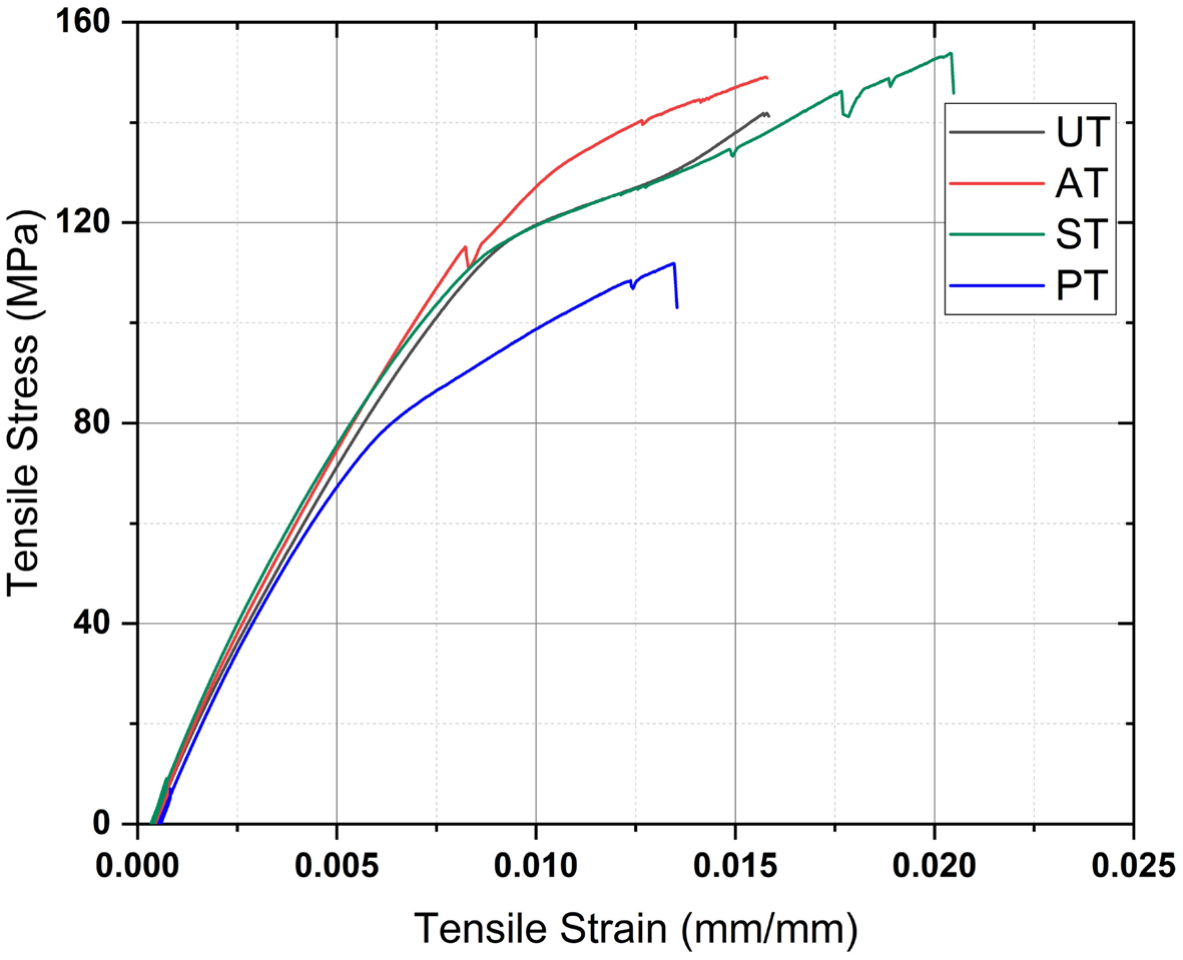
Tensile stress–strain curve of UT, AT, ST and PT composites.

Tensile characterization is essential for evaluating the strength and elongation behavior of materials under linearly applied forces. The incorporation of *Helicteres isora* fibers into PLA matrices introduces a natural reinforcement that can significantly impact the tensile properties of the resulting composites. The tensile behavior of UT, AT, ST and PT composite specimens are illustrated in Fig. 8. Tensile properties of Raw *Helicteres isora* fiber were determined as per ASTM D3822 standard. The tensile properties of raw fiber, neat PLA, UT, AT, ST and PT composites are listed in Table 4. Based on the obtained results, the Methacryl silane-treated *Helicteres isora* fiber-reinforced PLA composite (ST) exhibited the highest tensile strength, which is 153.15 ± 0.65 MPa. This is attributed to the bipolar coupling action of Silane, establishing a firm compatibility between the *Helicteres isora* fiber and the PLA matrix. Specimen ST has shown the highest strength and better modulus among all alternatives. The ST specimen exhibited lower brittleness, resulting in a lower tensile modulus (15.19 ± 0.06 GPa) compared to the modulus of PT specimen (15.85 ± 0.35 GPa), which had higher brittleness than other specimens. This argument is well supported by the failure mechanisms discussed in the upcoming Sect. 3.2.2.

**Table 4:** Tensile properties of raw *Helicteres isora* fiber, neat PLA, UT, AT, ST and PT composites.

Particulars	Tensile strength (MPa)	Tensile modulus (GPa)	Elongation at break (%)
Raw *Helicteres isora* fiber	274.98 ± 27.05	6.77 ± 1.20	1.93 ± 0.33
Neat PLA	34.65 ± 4.31	1.93 ± 0.07	3.50 ± 0.50
UT	148.05 ± 5.66	14.10 ± 0.18	1.66 ± 0.07
AT	152.32 ± 3.21	14.78 ± 0.27	1.91 ± 0.34
ST	153.15 ± 0.65	15.19 ± 0.06	2.27 ± 0.12
PT	108.99 ± 2.84	15.85 ± 0.35	1.70 ± 0.35

The tensile strengths of some established natural fiber-reinforced PLA composites are compared with *Helicteres isora* fiber-reinforced PLA composite in Table 5. The strength and modulus of *Helicteres isora* fiber-reinforced PLA composite are superior to many other natural fiber-reinforced PLA composites.

**Table 5:** Comparison of Tensile strength and moduli of PLA-based natural fiber composites.

Fiber + polymer	Tensile strength (MPa)	Tensile modulus (GPa)	References
Jute + PLA	152.00	5.30	^35^
Bamboo + PLA	72.00	2.60	^36^
Flax + PLA	60.00	2.00	^37^
Kenaf + PLA	76.56	3.17	^38^
Sisal + PLA	65.00	4.50	^39^
Banana + PLA	54.00	–	^40^
*Helicteres isora* + Epoxy	175–215	12.60	^16^
*Helicteres isora* + PLA (ST)	153.15	15.19	Present study

#### Failure mechanisms in tensile test

**Figure 9:**
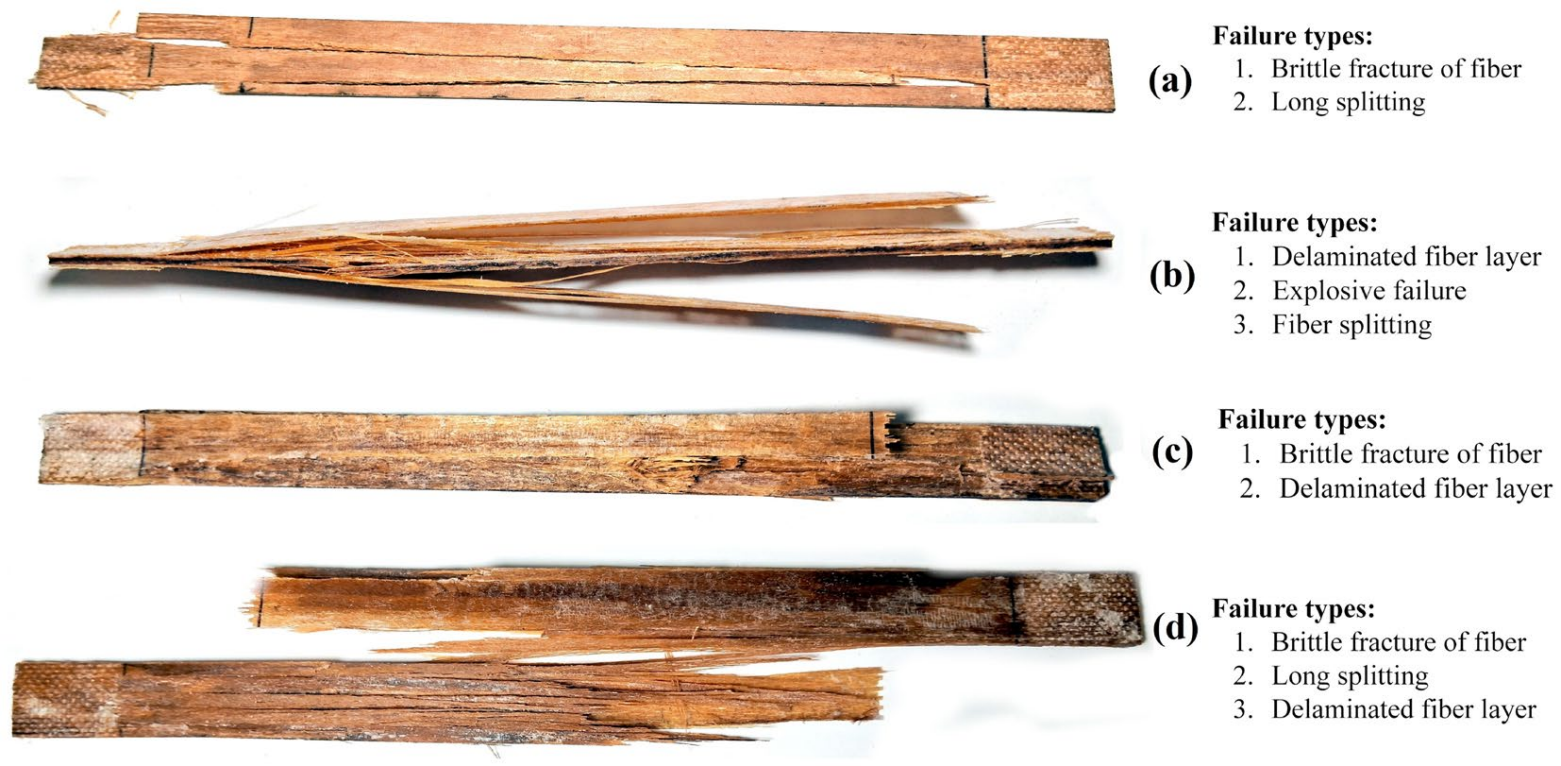
Tensile Failure mechanisms in (**a**) UT, (**b**) AT, (**c**) ST and (**d**) PT composites.

Failure mechanisms in natural fiber-reinforced polymer composites can result from various factors and interactions between the polymer matrix and the reinforcing natural fibers. A few of them are matrix cracking, fiber pullout, fiber fracture, interfacial debonding, and delamination of fiber layers. *Helicteres isora* fiber is a moderate to high modulus fiber with moderate to high tensile strength. According to ASTM D3039 testing method, failure modes in high-modulus fiber include explosive failure, long splitting along the fiber direction, and delamination of fiber layers. (a)–(d) shows the failure modes of UT, AT, ST and PT tensile specimens. UT specimen shows splitting and brittle fiber fracture during the tensile test. The explosive type of failure within the specimen's gauge length was observed in AT. This mode of failure is due to the high modulus of alkali-treated fiber. There are visible signs of fiber splitting, which suggests that the individual fibers within the composite split apart during the failure, and the delamination occurred at the edge. The composite ST tensile specimen has shown smaller delamination of the fiber-matrix interface in the fractured surface; this shows that the ST specimen has lower brittleness than its counterparts. Specimen of PT composite has shown broom like failure; this particular mode of failure was attributed to the occurrence of fiber splitting at various locations within the specimen gauge length. Before the ultimate and complete failure of the specimen, there was a process of delamination taking place at the surface layer. This delamination, or separation of layers, was a crucial precursor to the subsequent failure^40–43^ (Fig. 9).

**Figure 10:**
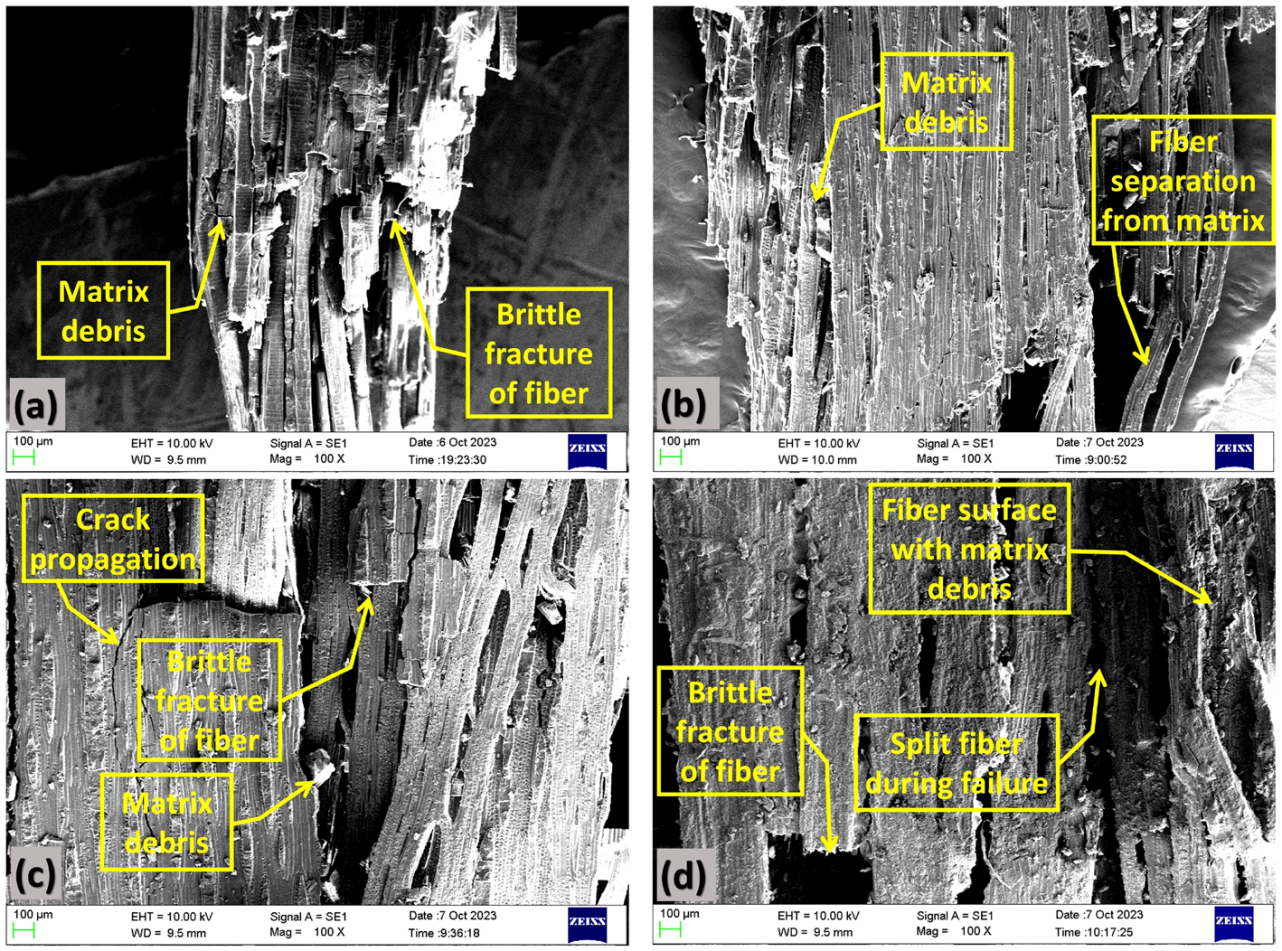
SEM image of a fracture surface of (**a**) UT, (**b**) AT, (**c**) ST and (**d**) PT specimen.

The SEM image of a fractured surface of the UT composite specimen shown in Fig. 10a depicts the brittle fracture of the *Helicteres isora* fiber. Which in turn illustrates the low ductility of the composite. SEM image of the fractured end of the AT specimen, as illustrated in Fig. 10b, shows the fiber splitting and matrix debris of the fiber-matrix interface. The surface of the fibers exhibited the presence of matrix debris, indicating that the matrix material had separated from the fibers during the course of the failure. SEM image of the ST composite specimen’s fractured section shown in Fig. 10c highlighted the crack formation during the failure and the brittle fracture of fibers perpendicular to their direction. It is observed that the specimen exhibited improved fiber-matrix adhesion, as evidenced by the limited occurrence of fiber delamination in a small segment in comparison to the other test specimens. PT composite specimen’s fractured surface SEM image is shown in Fig. 10d, which revealed the fibers themselves displayed characteristics of brittle fracture, suggesting that they had fractured with limited plastic deformation. This brittle failure behavior of the fibers is a key aspect of the overall failure mechanism.

#### Flexural properties of the composites

**Figure 11:**
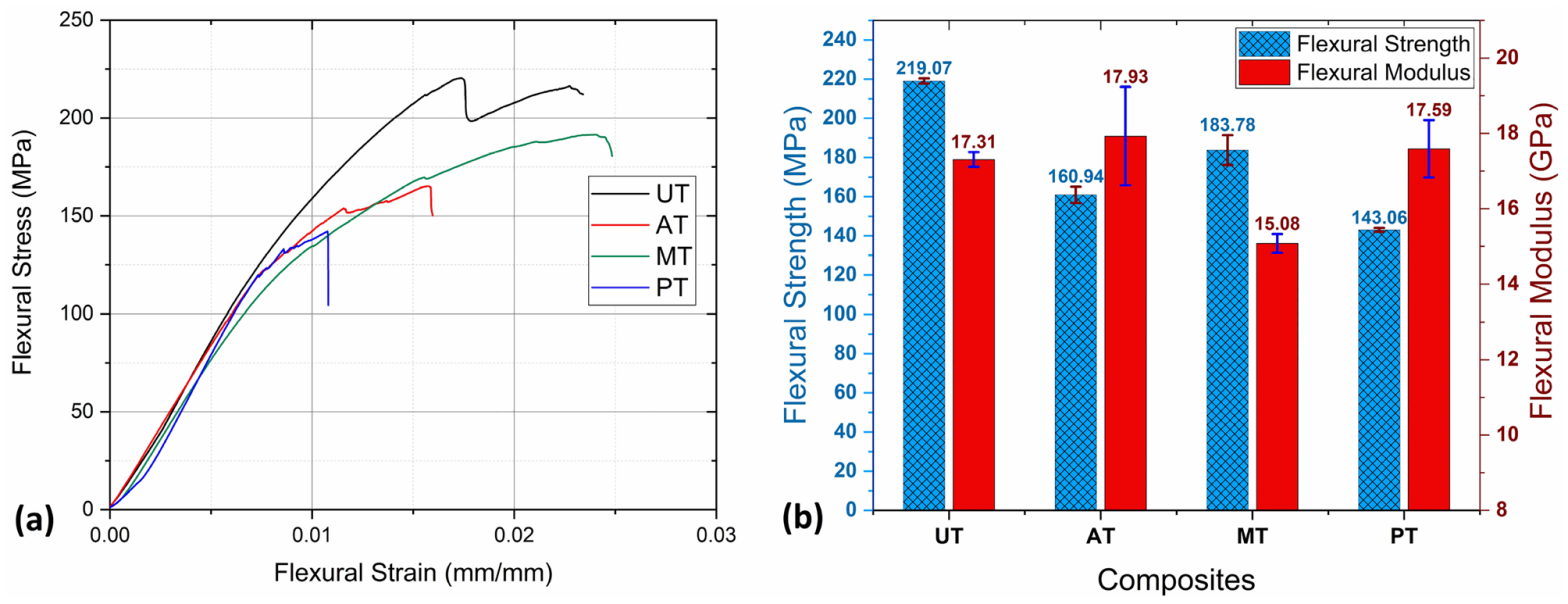
(**a**) Flexural stress–strain curve, (**b**) Flexural strength and moduli of UT, AT, ST and PT composites.

The flexural behavior and properties are illustrated in Fig. 11a,b. One of the key observations from this illustration is that the flexural strength of the untreated *Helicteres isora* fiber composite surpasses that of the chemically treated fiber composites. This finding can be attributed to the specific changes that occur in the fibers as a result of the chemical modification process. When fibers are chemically treated, there are instances where the resulting modification leads to an increase in the brittleness of the fibers. Brittleness implies that a material becomes more prone to fracture or failure without undergoing significant deformation. The increased brittleness in the chemically treated fibers causes reduced flexural strength; this is because, during a flexural test, materials that are more brittle tend to reach their limit and fail suddenly. Due to the increased brittleness of the fiber, alkali-treated fiber composite has shown lower flexural strength (160.94 ± 4.16 MPa) and higher flexural modulus (17.93 ± 1.30 GPa) than that of untreated fiber composite. Silane-treated fiber composite has shown moderate flexural strength (183.78 ± 7.62 MPa) and lower modulus (15.08 ± 0.25 GPa) due to its high ductile behavior as observed in tensile characterization.

### Thermal behavior of composites

**Figure 12:**
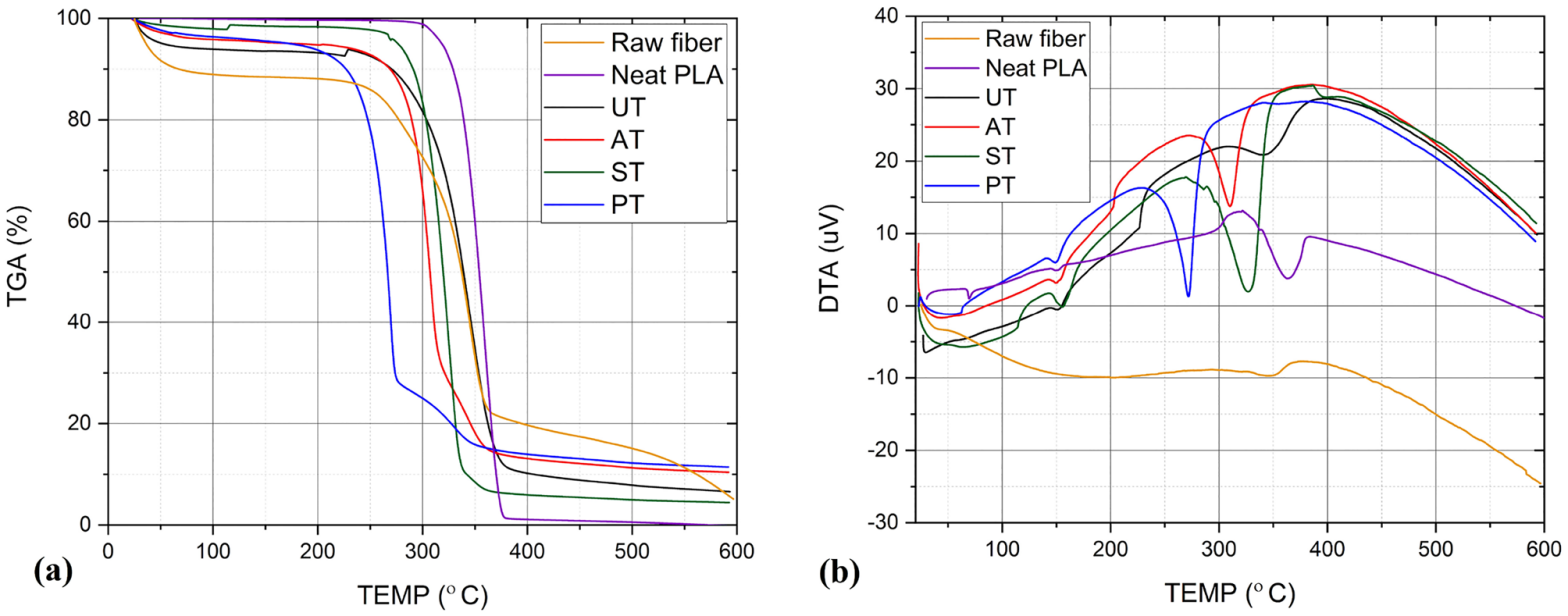
(**a**) TGA and (**b**) DTA curves of Raw fiber, neat PLA, UT, AT, ST and PT composite specimens.

The superimposed TGA and DTA curves of Raw *Helicteres isora* fiber, neat PLA, UT, AT, ST and PT specimens are shown in Fig. 12a,b, respectively. Up to 100 °C, the initial weight loss observed can be attributed to the evaporation of moisture and other low-temperature volatile compounds present in the specimens. Beyond this point, the curves for different specimens diverge, indicating variations in thermal stability. In raw fiber samples, the weight loss within 100 °C is more due to the higher moisture content of the fiber^44^. Neat PLA has the least weight loss before 100 °C because of its hydrophobicity and low moisture content. In the PT specimen, material degradation commenced at 225 °C. For the UT and AT specimens, this degradation process was slightly delayed, initiating in the temperature range of 250–270 °C. Notably, the ST specimen's curve exhibited a different behavior. In this case, degradation did not commence until 270 °C, and degradation ended in the temperature range of 340–350 °C. This observation indicates that the composite with silane-treated fibers exhibits higher thermal stability up to 270 °C compared to the other composite specimens. In other words, it can withstand higher temperatures without significant degradation or weight loss.

The DTA curves of composite specimens reveal both endothermic and exothermic reactions occurring during the heating process. The peaks, both exothermic and endothermic, along with their magnitudes, signify the thermal phase transformation of the composites. Endothermic reactions involve processes like the evaporation and melting of volatile compounds and polymeric materials. In contrast, exothermic reactions encompass chemical reactions and oxidative degradation. The upward peak in the DTA curve signifies the oxidative decomposition of the material. Notably, within the temperature range of 200–300 °C, there is an endothermic reaction associated with cellulose degradation. This is evidenced by the descending peak observed between 225 and 275 °C, indicating the degradation of cellulose present in the fiber. The weight loss of *Helicteres isora* fiber, with significant decomposition occurring within the temperature range of 225–375 °C^45^. This decomposition primarily stems from the breakdown of hemicellulose, cellulose, and lignin present in the fiber. The decomposition of natural fibers initiates with hemicellulose, followed by cellulose, lignin, and ash. Hemicellulose decomposition typically commences early, around at 220 °C, attributed to its chemical composition featuring a random amorphous structure with minimal strength, thus rendering it readily hydrolyzed^46^. As for the degradation of lignin within the fiber, it initiates at a temperature of 400 °C and persists until reaching 600 °C. The gradual degradation of residual lignin is signified by the slope of the curve after 400 °C^47^.

The PLA polymer shows an initial peak at 70 °C, which exhibits the glass transition temperature of PLA. The curve also exhibits a melting point at 200 °C and begins to degrade at 300 °C, ending at 380 °C^48^. Each developed composite shows similar behavior in the TGA and DTA curves with a slight shift in the degradation peaks due to the partial removal of hemicellulose and varied thermal stability of the fiber upon chemical treatments. DTA curves of AT, ST and PT specimens show that the major oxidative degradation of material started at 310 °C, 335 °C and 275 °C, and ended after 350 °C, 375 °C and 325 °C respectively. Overall, the results from the DTA curve demonstrate the composite materials' ability to maintain thermal stability at elevated temperatures. As per the results obtained, Silane-treated fiber-reinforced PLA composite shows a more stable behavior before degradation up to 275 °C than all other specimens.

## Conclusions

Based on the results obtained from the physical, mechanical and thermal characterizations carried out on the *Helicteres isora* fiber-reinforced PLA biodegradable composites prepared by using a combined fabrication technique, which is solution casting followed by compression moulding, and based on the literature referred and the correlation of results with the previously published results, following conclusions are drawn.

- Specimen PT exhibited the lowest void fraction among all the prepared specimens. ST specimen exhibits a lower void content than the UT and AT specimens and a slower water absorption tendency than all other specimens. Lower void content in treated (ST) composites resists the diffusion of water molecules, thereby reducing water absorption.
- In terms of tensile performance among the UT, AT, ST, and PT specimens, it is evident that the Methacryl silane-treated (ST) composite displayed improved tensile properties by 7% above UT specimens, which indicates that the fiber-matrix bonding has been enhanced by Silane treatment on the fiber surface. Analyzing the flexural behavior of all specimens under loading conditions reveals that the UT specimen exhibits the highest strength, followed closely by ST. Conversely, the PT specimen demonstrates the lowest flexural strength but exhibits the highest flexural modulus.
- Assessing the thermal stability of the materials, it becomes clear that the ST specimen outperforms the others in terms of stability. TGA shows that the ST composite maintains its structural integrity and exhibits a higher decomposition temperature, indicating greater resistance to thermal degradation. Consequently, ST is the most thermally stable composite among its counterparts.
- Considering the collective evaluation of mechanical, physical, and thermal characteristics across the prepared composites, the Methacryl-silane-treated *Helicteres isora* fiber-reinforced PLA composite (ST) clearly stands out as the most favorable choice for potential applications in lightweight structural components and interiors of automobile.
- The developed composite is fully bio-based and bio-compostable; hence, it is suitable for use in eco-friendly packaging where strength and sustainability are required. The composite developed is useful in food article packaging and storage applications upon conducting toxicity tests and confirming the safety of the composites. Its biodegradability and other useful properties it makes a sustainable alternative to food packaging and storage materials.

## Data Availability

The data generated and analyzed during the current study are not publicly available, but are available from the corresponding author on reasonable request.
